# Circulating 16S RNA in Biofluids: Extracellular Vesicles as Mirrors of Human Microbiome?

**DOI:** 10.3390/ijms21238959

**Published:** 2020-11-25

**Authors:** Veronica Ricci, Davide Carcione, Simone Messina, Gualtiero I. Colombo, Yuri D’Alessandra

**Affiliations:** 1 Unit of Immunology and Functional Genomics, Centro Cardiologico Monzino—IRCCS, 20138 Milan, Italy; vricci@ccfm.it (V.R.); simone.messina1@studenti.unimi.it (S.M.); gcolombo@ccfm.it (G.I.C.); 2 Dipartimento di Medicina Clinica e Chirurgia, Università degli Studi di Napoli Federico II, 80138 Napoli, Italy; 3 Unit of Laboratory Medicine, Centro Cardiologico Monzino—IRCCS, 20138 Milan, Italy; davide.carcione@gmail.com

**Keywords:** 16S, plasma, extracellular vesicles, microbiome

## Abstract

The human body is inhabited by around 10^13^ microbes composing a multicomplex system, termed microbiota, which is strongly involved in the regulation and maintenance of homeostasis. Perturbations in microbiota composition can lead to dysbiosis, which has been associated with several human pathologies. The gold-standard method to explore microbial composition is next-generation sequencing, which involves the analysis of 16S rRNA, an indicator of the presence of specific microorganisms and the principal tool used in bacterial taxonomic classification. Indeed, the development of 16S RNA sequencing allows us to explore microbial composition in several environments and human body districts and fluids, since it has been detected in “germ-free” environments such as blood, plasma, and urine of diseased and healthy subjects. Recently, prokaryotes showed to generate extracellular vesicles, which are known to be responsible for shuttling different intracellular components such as proteins and nucleic acids (including 16S molecules) by protecting their cargo from degradation. These vesicles can be found in several human biofluids and can be exploited as tools for bacterial detection and identification. In this review, we examine the complex link between circulating 16S RNA molecules and bacteria-derived vesicles.

## 1. The Human Microbiome

Humans host around 10^13^ of microorganisms composing the “human microbiome” and populating different niches of the human body, thus playing important roles in the maintenance of homeostasis of several anatomical sites.

The term microbiota was first used by Lederberg and McCray to describe the importance of the microorganisms residing in the healthy human body and during disease. The term “human microbiota” describes the types of bacteria and other microorganisms that live inside or on the human body, while the sum of all genomes of these symbiotic microorganisms is defined as “metagenome” [1].

The first studies on this subject started with Antonie van Leeuwenhoek—considered the father of microbiology. He gave the first description of protists and bacteria living in different environments [2]. Today, these differences have been widely explored, and the focus has shifted towards the mechanisms regulating variations of microbial composition under both physiological and pathological conditions. The majority of human microbes reside in our gastrointestinal tract [3]. Among the several different bacterial species within the gut microbiota, the most represented are the *Firmicutes* and *Bacteroidetes* phyla, performing different functions such as protection against pathogens, metabolization of dietary compounds, vitamins production, and immune modulation [4]. Over the last few years, studies on the role of the microbiota in organisms’ homeostasis have gradually increased and the attention of researchers focused on the role of these symbiotic microorganisms in previously overlooked organs, such as the lungs, vagina, and blood [5].

## 2. The Microbiome as a Gateway to Detect the Microbiota Composition: 16S Sequencing

The constant increase in the number of studies on the microbiota and its interactions with the human organism is largely due to the use of new technologies involving sequencing analysis. In fact, before the adoption of next-generation sequencing (NGS) in this field, the detection of microorganisms from different human districts was conducted by culturing methods, but, beyond being time-consuming, these tools were greatly hampered and unsuccessful due to “unculturable” bacteria [6].

**Figure 1:**
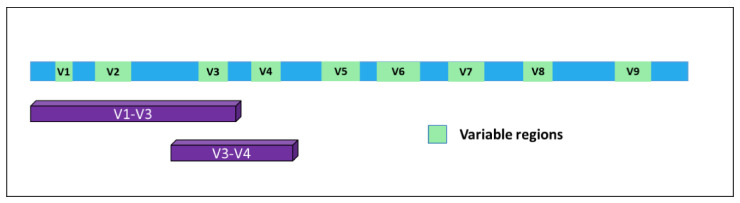
Structure of the 16S gene. The nine variable regions are depicted in green. Purple bars indicate the portions of the gene mostly used for bacterial classification upon PCR-based amplification and sequencing.

In time, it became evident for the necessity to identify markers allowing for the detection of microorganisms in a particular substrate and, together, their identification at the species level with high confidence. A very good response to this unmet need was represented by a specific class of RNAs. In bacteria, the 5S, 16S, and 23S ribosomal RNAs are organized in a gene cluster which is expressed as a single operon. The size, sequence, and secondary structures of these three rRNA genes are highly conserved between different bacterial species. In particular, the 16S rRNA [7] originates from a gene of about 1500 bp present in all bacteria and contains nine hypervariable and species-specific regions (V1—V9) flanked by highly conserved and well-known portions of the genome. Thus, by using specific PCR primers, it is possible to amplify parts of the gene containing both the constant and the variable sequences, which are very useful for microbial classification (Figure 1). Indeed, this gene has been used for phylogenetic studies for many years and is considered the gold standard for microbiome detection and classification [8].

The first example of 16S amplification and sequencing using the Sanger method was presented in 1990 but was soon followed by the advent of high throughput sequencing [9,10], which represented a turning-point and a revolution in terms of costs and speed of detection, boosting the development of microbiome-based studies. Indeed, Sanger sequencing following PCR amplification was successful in identifying bacterial strains in monomicrobial infections, for example, outperforming conventional culture methods or diagnostic body fluid markers, but failed to identify unique molecular targets in polymicrobial infections. This is due to the intrinsic property of the Sanger method, which can be performed only on DNA molecules with the same sequence, while NGS allows for massive parallel sequencing of many different DNA templates.

In 2007, the Human Microbiome Project (HMP) was started to use next-generation sequencing methods to identify the abundance, diversity, and functionality of the microorganisms that live in different sites of the human body, thus generating a reference database for subsequent comparative analyses [11]. With this aim, 16S-focused investigations were implemented. The 16S analysis typically starts with the amplification of specific variable regions of the 16S rRNAs (usually V3—V4), to be massively sequenced in parallel; then, the obtained sequences are clustered into Operational Taxonomic Units (OTUs) [12]. These are defined according to their similarity to each other based on a threshold, usually defined as a sequence similarity of at least 97%. In time, several bioinformatics pipelines have been implemented to analyze the results of 16S sequencing, such as QIIME (Quantitative Insights Into Microbial Ecology, http://qiime.org/) [13] and MOTHUR (https://mothur.org/) [14], which were specifically designed for examining microbial communities.

The two principal parameters describing the complexity of microbiota in a definite environment are the α and β diversity. Alpha diversity describes the richness and evenness, i.e., the number of different organisms and the homogeneity of their distribution within a sample. Beta diversity is a measure of absolute or relative overlap in taxa shared between samples. There is a wide range of microbial β diversity in the microbiota between individuals since particular species could be widely abundant in some individuals and may be minimally represented in others [15]. In particular, more specific indexes belonging to both alpha and beta metrics are often used during the assessment and classification of bacterial communities. Among the most used there are the Shannon-Weaver, the Simpson, the Jaccard, and the Bray-Curtis, which quantify the taxonomic dissimilarity. Since all diversity indices have specific biases, they must be selected appropriately [16,17].

## 3. The “Healthy” Microbiome

The microbiota of each individual is mostly acquired from the mother at birth, thus starting the bidirectional dialogue between microorganisms and the host, which will continue for the entire existence. The microbial composition changes during the individual’s growth and becomes relatively stable within a few years, although it is susceptible to changes caused by external stimuli and aging [18]. In the first 2 years of life, the composition of the gut microbiota changes significantly but gradually, with an increase in both stability and diversity. Several factors contribute to this development, such as eating habits, antibiotics and probiotics intake, and maternal diet [19].

Changes in prokaryotic populations can also be observed during pregnancy, as shown by Aagaard et al. in a cross-sectional study analyzing the vaginal microbiome in 24 pregnant women [20]. Their results showed that bacterial diversity and richness of analyzed subjects were reduced in comparison to nonpregnant women. Besides pregnancy, multiple factors, such as smoking, diet, sport, lifestyle, age, social conditions, and the environment, can influence the microbiota composition in the lung, gut, skin, and vagina [21,22,23,24]. Benedict et al. showed that, after 2 days of partial sleep deprivation, healthy subjects presented a variation in gut microbiome diversity, with an increase in *Firmicutes:Bacteroidetes* ratio, a higher presence of *Coriobacteriaceae* and *Erysipelotrichaceae*, and a lower abundance of *Tenericutes*, when compared with individuals experiencing normal sleep [25]. In another setting, Shively et al. conducted a study on 31 female monkeys undergoing different diets: the consumption of a Mediterranean diet was associated with a high abundance of *Lactobacilli* in the mammary gland and an elevation of bile acid metabolites, showing that, besides the expected effects on the gut, dietary habits can modulate the microbial composition of different organs [26].

## 4. Microbiota and Disease

Since different anatomical sites of our organism possess a specific microbiota, it is quite obvious that perturbation in its composition could lead to and/or take part in several human maladies. Indeed, many studies reported an association between dysbiosis and the onset and/or progression of several diseases [27]. For example, alterations in lung microbial communities are involved in different respiratory syndromes. Sequencing analyses conducted on lung tissue samples obtained from patients with chronic obstructive pulmonary disease (COPD) or cystic fibrosis and healthy controls showed different microbiota composition in the three groups [28]. Similar studies showed that the respiratory microbiota was different during COPD exacerbations compared to periods of clinical stability, proving that the modulation of the microbiota is also related to the stage of the pathology [29].

Associations between gut microbiota and cardiovascular diseases (CVD), such as atherosclerosis, coronary heart disease (CAD), and myocardial infarction have been widely studied [30]. Zhu et al. characterized a signature in the gut microbiota of patients affected by CAD [31]. The analysis of stool samples from 70 patients with CAD and 98 healthy controls showed a lower abundance and species diversity in the CAD group, with a prevalence of *Faecalibacterium* in healthy subjects and *Escherichia—Shigella* and *Enterococcus* in the CAD group.

Similarly, in the cancer setting, microbiota-focused studies evidenced the involvement of Helicobacter pylori in gastric-adenocarcinoma [32], leading to the classification of this microorganism as a class 1 human carcinogen. Indeed, cancer is characterized by the complex interaction between different molecular pathways, and the microbiota can influence all these mechanisms by secretion of bacterial metabolites, capable of interfering with cell proliferation or apoptosis, thus contributing to disease onset and progression [33]. Of note, dysbiosis has been associated with a high risk of cancer development, since microbiota composition can influence and trigger the onset of several alterations that seem to be involved in tumorigenesis. This phenomenon has been observed in colorectal cancer and breast and hepatocellular carcinoma [34].

Interestingly, the human microbiota has been proposed as a key factor also in the pathophysiology of neuronal maladies such as Parkinson’s or Huntington’s disease, amyotrophic lateral sclerosis, and autism spectrum disorders (ASD) [35]. A meta-analysis conducted in 2019 on the alteration of gut microbiota in autistic and health subjects revealed a lower abundance of *Bifidobacterium*, *Enterococcus*, *E.coli*, and *Bacteroides* and a higher abundance of *Lactobacillus* in children with ASD [36]. The authors concluded that an altered balance between the levels of “beneficial” bacteria combined with the deregulated levels of “detrimental” bacteria could contribute to ASD symptoms.

## 5. Bacteria in the Blood: To Be or Not to Be? Circulating 16S Detection

As already mentioned, 16S detection by sequencing is considered a hallmark of bacterial presence and a way to assess microbial diversity since the advent of NGS-based tools. Recently, though, the attention of several groups has been drawn to the detection of 16S RNA in “germ-free” niches, including the blood of healthy subjects. Indeed, blood has traditionally been considered devoid of microbial presence and the detection of microorganisms in this biofluid has long been considered a sepsis index.

Nikkari et al. showed that the blood of healthy human subjects contained bacterial 16S, but the mechanisms underlying its presence were not assessed [37]. One explanation involves the transmission from the mother before birth or translocation from other sites during the normal lifecycle. The human blood-microbiota appears to be principally composed of the phylum *Proteobacteria* followed by *Actinobacteria, Firmicutes*, and *Bacteroidetes*, although variability was observed across the different investigations [38]. Paisse et al. analyzed the microbiome composition in separate blood fractions [39]. They extracted DNA from whole blood, buffy coat, and red blood cells (RBC) fractions of 30 young and healthy volunteers and performed quantitative PCR analysis and sequencing of V3—V4 hypervariable regions of the 16S rRNAs for taxonomic classification. The highest abundance was observed in the buffy coat (93.74% of bacterial DNA), followed by RBCs (6.23%) and plasma (0.03%), with the RBC fraction showing a higher bacterial diversity than the other two components. Interestingly, in the blood, they found mostly *Proteobacteria* (more than 80%) and *Actinobacteria* (6.7–10% depending on the fraction), at variance from the phyla predominant in the gut (*Firmicutes* and *Bacteroidetes*) [40].

## 6. Circulating 16S in a Disease Context

As previously said, numerous studies analyzed the role of the microbiome in relation to the onset of different pathologies, most of them focusing on the intestinal microbiome. Lately, though, an increasing number of researchers investigating connections between dysbiosis in the blood microbiome and human disease. In a work from 2011, Amar et al. showed, for the first time, that the blood microbiome composition might predict the onset of diabetes, in a 6–9-year follow-up [41]. About a year later, the authors performed 16S qPCR on the blood of individuals without CVD at baseline and found a decrease in blood bacterial DNA and an increase of *Proteobacteria* in subjects who suffered from cardiovascular complications during the follow-up [42]. In a subsequent work in 2014 on circulating human microbiome in CVD subjects, Dinakaran et al. found an increase in microbial diversity and bacterial DNA concentration in the blood of patients [43]. Here, they observed a predominance of *Actinobacteria,* while the most abundant phylum in healthy subjects was *Proteobacteria*.

In a different study, Lelouvier et al. investigated the association between blood microbiota and the onset of liver disease [44]. The authors performed both 16S qPCR and sequencing to unveil the relationship between blood bacterial population composition and liver fibrosis in obese patients. The results showed higher concentrations of 16S in the blood of patients with fibrosis than in healthy subjects, thus identifying a specific microbial cluster associated with liver fibrosis that was suggested as a biomarker for its early detection. 

Qian et al. in 2018 investigated the possible association between blood microbiota alteration and Parkinson’s disease [45]. The taxonomic diversity was assessed by performing 16S sequencing and some genera resulted associated with the pathology, with *Cloacibacterium*, *Isoptericola*, *Paludibacter*, and *Saccharofermentans* genus showing a correlation with disease duration.

More recently, a manuscript from Hammad and co-authors described the profiles of circulating microbial DNA in patients with rheumatoid arthritis (RA) in comparison with patients affected by ankylosing spondylitis (AS) or psoriatic arthritis (PA) and healthy control subjects [46]. Bacterial community members were identified by sequencing of the 16S rRNA variable region 4 in all samples. At the phylum level, the blood microbiome was predominated by four phyla, i.e., *Proteobacteria*, *Firmicutes*, *Bacteroidetes* and *Actinobacteria*, supporting the notion of a core blood microbiome as reported in previous publications [39,42,44,47]. In 2016, Santiago and coworkers presented an investigation assessing serum microbial composition in patients with and without ascites [48]. They performed 16S rDNA high-throughput sequencing, evidencing complex and specific microbial communities in serum and ascitic fluid of patients with cirrhosis. Interestingly, sera obtained from healthy controls resulted in an almost complete absence of bacteria. Of note, the authors indicated the presence of an unknown phylum belonging to *Cyanobacteria* in the serum of patients with ascites.

## 7. Source of Circulating 16S RNA: Are We Really Looking at Bacteria in the Blood?

The source of circulating 16S molecules has been discussed for many years. Some researchers hypothesized that the bacteria in the blood originated from gastro-intestinal tract leakage, but it has also been suggested that they could derive from skin or the oral tract and that they diffuse in blood when these protective barriers are compromised [38]. An additional hypothesis, though, could be represented by extracellular bacterial vesicles. Indeed, both Gram-negative and Gram-positive bacteria can release spherical membrane vesicles (MV) derived by the cell membrane and containing several molecules involved in different functions [49]. To date, the studies on the biogenesis, structure, and function of MV in Gram-positive bacteria are just beginning, while detailed studies have been conducted on Gram-negative microbes.

To better understand the structure and function of these vesicles, it is worth making a brief introduction to the composition of the bacterial envelope. The cellular structure of Gram-negative bacteria is characterized by a double cell membrane divided by a periplasmic space containing a layer of peptidoglycan. The outer membrane (OM) is quite peculiar, as it is composed of an outer leaflet consisting of lipopolysaccharide (LPS) and an inner leaflet consisting of phospholipids. The presence and distribution of LPS have a tremendous impact on the ability of the bacteria to survive in harsh environments, as they regulate the impermeability to hydrophobic compounds such as antibiotics and detergents. Differently, the inner membrane is composed of a phospholipid bilayer and proteins and encloses the contents of the bacterial cell [50,51].

The outer membrane vesicles (OMV) are spherical structures of 20–300 nm produced by Gram-negative bacteria, and derive by the blebbing of the outer plasmatic membrane, being composed of the outside membrane and periplasmic material. The detachment of the outer membrane, in pathogenic and nonpathogenic bacteria, is not limited to a stochastic process of fragmentation and splitting but is a finely controlled process occurring during the normal growth of bacteria and in which several environmental factors are involved [52]. Different triggers of vesicle biogenesis were identified in Gram-negative bacteria, involving alteration in peptidoglycans structure, accumulation of LPS, and enrichment of the outer membrane with phospholipids. Moreover, several pieces of evidence showed that alteration of the microbial structure caused by various environmental factors, such as antibiotics or temperature variations, may cause vesicle accumulation [53].

Differently from Gram-negative bacteria, the biogenesis and composition of extracellular vesicles in Gram-positive bacteria remain quite elusive. The protein composition of extracellular vesicles (EVs) produced by the Gram-positive bacterium *Staphylococcus aureus* was investigated in 2009. EVs were reported to be similar in size to their Gram-negative counterparts (20–100 nm in diameter), while their cargo included a variety of proteins that are important for survival and virulence [54]. Since Gram-positive are characterized by a thick peptidoglycan cell wall outside of the cell membrane, the production and release of MV is a highly regulated and complex process. To date, three different mechanisms of EVs release have been proposed: (1) the vesicles may be pushed through the wall by turgor pressure, and their size could be affected by cell wall pore size or thickness; (2) cell wall modifications could be induced by specific modifying enzymes, thus facilitating its loosening and triggering EV release; (3) specific channel-like structures could facilitate EVs passage through pores, guided by tubulin [55].

Regardless of their source, the cargo of bacterial EVs displays quite a heterogeneous arrangement, including inner-membrane, periplasmic and cytoplasmic components, genetic material (DNA/RNA), toxins, and also factors involved in antibiotic resistance. OMVs are essential for different functions such as cell-to-cell communication, the formation of biofilms, bacterial infections, and the transfer of proteins and genetic material [56].

## 8. Bacteria-Derived Vesicles and 16s RNA 

As proposed above, one possible explanation of 16S detection in the absence of bacteria is the fact that the gene can be transported by extracellular structures. Here we present the current knowledge-based on existing literature. The main features of these works are presented in Table 1.

One of the first papers describing the presence of 16S in bacteria-derived vesicles in a disease context was published in 2013. The authors used a colitis mouse model to evaluate the proportion of bacteria and bacteria-derived EVs in the large intestine, small intestine, and small intestinal fluids. Interestingly, when comparing the bacterial composition of stool samples with stool-derived EVs in the large intestine, they found a large disproportion, suggesting that intestines are host to a great diversity of bacteria, but that not all these bacteria can produce EVs. A similar situation was observed in the small intestine. In their conclusions, the authors claimed to be the first to use EV-derived 16S detection to analyze the composition of gut microbiota-derived EV, introducing a great leap towards a better understanding of complex microbial populations, particularly of those microbes which were either difficult or impossible to cultivate [57].

Since that first study, numerous research groups followed suit, exploiting vesicle isolation in combination with 16S sequencing to evaluate microbial composition in different contexts.

In 2016, Yoo and coworkers conducted 16S rRNA-based analysis in bacteria-derived extracellular vesicles in the urine of pregnant and nonpregnant women [58]. In their work, the authors amplified and sequenced by NGS the V1—V3 region of 16S after differential centrifugation-based EV isolation from the urine of 73 non-pregnant and 74 pregnant women. When the two groups were compared, the levels of 13 microbial taxa exhibited significant differences. In particular, *Bacillus* was the taxon that was more significantly enriched in pregnant women than in non-pregnant women (mean composition = 45.61% vs. 0.12%). On the opposite, *Pseudomonas* was the taxon that was more enriched in nonpregnant women than in pregnant women (mean composition = 14.23% vs. 4.09%) together with *Lactobacillus* (8.44% vs. 1.61%). Moreover, the authors showed that, in a few specific cases, EVs derived from *Ureaplasma* were more frequently detected in the urine of women who underwent preterm delivery, and *Ureaplasma, Fusobacterium,* and *Sneathia* were increased in the urine of preterm-delivered women with premature babies who had pneumonia, meningitis or urinary tract infection.

In a subsequent paper, Choi and colleagues assessed the clinical significance of *H.pylori* EVs in gastric juices [59]. They enrolled healthy controls and patients with gastric cancer, separated EVs by density-gradient ultracentrifugation of the gastric juices collected by endoscopy or surgery, and then performed next-generation metagenomic sequencing of the microbial 16S rDNA gene. The hypervariable portion of 16S was used to calculate the Chao1 index, an estimator based on abundance. Bacterial EVs showed that, at the genus level, several microorganisms had a differential abundance when comparing healthy controls with cancer patients. In particular, *Streptococcus*, *Gemellaceae, Oribacterium*, and *TM7-3* were increased in EVs from gastric cancer patients compared to healthy controls. Interestingly, the authors evidenced a very high penetrating ability of *H. pylori* vesicles into stomach epithelial cells.

Circulating EVs and 16S sequencing were used in a completely different setting by Park and co-authors, investigating gut microbiota contribution to brain dysfunction in a mouse model of Alzheimer’s disease (AD) [60]. In particular, sequencing of V3—V4 hypervariable regions of 16S rDNA was performed after isolation and boiling of serum EVs. The results showed five phyla (*Firmicutes*, *Proteobacteria*, *Bacteroidetes*, *Actinobacteria* and *Saccharibacteria*) as the most represented, comprising nearly 95% of the identified bacteria in both wild-type and AD-mice. Notably, the proportion of *Firmicutes* increased from 34.7 to 57.5% in AD-mice, whereas *Proteobacteria* decreased from 30.5 to 20.7%. The microbial composition of AD-mice EVs was altered, emphasizing the importance of the relationship between microbiota and AD. Interestingly, the microbiota represented in EVs matched the gut microbiota reported in previous studies, an important indication of the possible exploitability of circulating 16S as a marker of bacterial presence in the intestine.

Bacteria-derived extracellular vesicle seems to play important roles also in allergies, as shown by Samra and colleagues in 2018 in their investigation on biomarkers for monitoring allergic airway diseases in children [61]. Indeed, 118 subjects divided into four groups (chronic rhinitis, CR, allergic rhinitis, AR, atopic asthma, AS, and healthy controls) were enrolled to collect urine samples as a substrate for EVs isolation, followed by DNA extraction and 16S-rDNA sequencing. Principle component analysis of the results showed that the samples from the CR, AR, and AS subjects clustered similarly, although with partial overlap with the controls. The major phyla detected in all samples were *Proteobacteria*, *Actinobacteria*, *Firmicutes*, *Bacteroidetes* and *Cyanobacteria*, which together constituted 96.5–97% of the community composition. *Actinobacteria* were significantly enriched in AS and CR groups.

Another substrate for vesicle isolation and 16S-based microbiome investigation is sputum [62]. In 2019, Philley and associates sequenced the 16S rDNA V4 region in serum-derived extracellular vesicles and cultured expectorate from healthy controls, women with nontuberculous mycobacterial lung disease, and women with both nontuberculous mycobacterial lung disease and breast cancer. The microbiome community was dominated by *Streptococcus*, *Haemophilus*, *Veillonella*, *Neisseria*, *Prevotella*, *Fusobacterium*, *Bacteroides*, *Allistipes*, *Faecalibacterium* and *Staphylococcus*. Interestingly, many of the identified genera are associated with the development of various lung and oral cavity diseases including malignancies.

The following year, similar work was conducted by Lee and colleagues in patients suffering from biliary tract cancer, benign inflammation, and in a control group of healthy subjects [63]. The authors isolated EVs from plasma using differential centrifugation, followed by DNA extraction, amplification, and sequencing of the V3—V4 hypervariable regions of 16S rDNA. *Proteobacteria, Firmicutes, Actinobacteria, Bacteroidetes,* and *Cyanobacteria* composed 94.7% of the identified OTUs in healthy subjects. These groups, though, covered 93.8% of the total OTUs in the biliary tract cancer individuals and 88.1% in the benign inflammation group. Interestingly, the authors observed that compositional differences of *Bifidobacteriaceae*, *Oxalobacteraceae Ralstonia, Pseudomonaceae* family, *Corynebacteriaceae Corynebacterium*, and *Comamonadaceae Comamonas* species significantly differentiated biliary tract cancer patients from healthy individuals. Thus, they were able to develop a biliary tract cancer prediction model merging these five variables with chronologic age and gender. This study was of great significance to underline the potential diagnostic value of vesicles-associated circulating 16S in the future.

Serum-derived bacterial vesicles were used also to isolate and sequence the V3—V4 hypervariable region of the 16S rDNA gene in psychiatric conditions settings by Rhee and co-authors [64]. They compared the serum microbiome composition of patients with bipolar disorder, major depressive disorder, and healthy controls. The study evidenced *Firmicutes*, *Proteobacteria*, *Bacteroidetes*, *Actinobacteria* and *Verrucomicrobia* as the most abundant taxa in all groups. A more thorough analysis evidenced that *Prevotella* 2 and *Ruminococcaceae* UCG-002 genera were significantly more prevalent in patients with the major depressive disorder than in either those with bipolar disorder or in healthy controls. Since these microbial genera are able to induce inflammatory disorders, and given that inflammation and immune dysregulation are at the base of several mood illnesses, the authors speculated their possible role in triggering depression and other mental conditions.

The most recent investigation regarding the use of extracellular vesicles-derived 16S for sequencing-based taxonomy identification was focused on the development of diagnostic models to differentiate ovarian cancer (OC) and benign ovarian tumor (BOT) [65]. In 2020, Kim and co-workers described the isolation of EVs from the serum of 166 patients with OC and 76 patients with BOT. Similar to almost all previous works, the authors amplified and sequenced by NGS the V3—V4 hypervariable regions of the 16S rDNA gene. The diagnostic model for OC was designed by randomly dividing the samples from each group into training and test sets in the ratio 2:1. The results showed a significant prevalence of *Acinetobacter* in the OC group. More importantly, this genus, together with age, and serum CA-125 levels (a protein marker of possible ovarian cancer presence) showed the best diagnostic performance to distinguish between OC and BOT.

## 9. Limitations and Functional Perspectives

A point of caution when interpreting these experiments is the possible contribution of contaminations. Indeed, contamination is one of the biggest issues in the microbiological field, concerning 16S-based classification, [66] particularly when very low amounts of bacteria and bacteria-derived molecules are involved [67]. In this setting, the presence of contaminants from the environment, laboratory reagents, and personnel involved in sample preparation could greatly affect the results of the investigations, leading to improper conclusions. Of interest, it has been shown that the timing of nucleic acid extraction is also important in longitudinal studies such as the one from Turner et al. [68], since different batches of the same extraction kits could result in the detection of different contaminants [69]. These examples clearly indicate that microbiological investigations should always include several negative controls to control for contaminants and produce robust and reliable results.

It must be also noted that this topic is in its infancy. Thus, the several limitations present in all the discussed investigations, with adequate sample size and strict assessment of possible contaminations being the most important issues, clearly indicate the need for additional studies to increase our knowledge on the real characteristics of the “circulating microbiome”. In particular, since the advent of NGS with the ability to generate huge amounts of data, the possibility of generating false-positive associations between the microbiome and human physiology and disease is high. Thus, experiments providing proof of functional relevance for both gut microbes and bacteria-derived vesicles in human health and pathophysiology are of utmost importance. For instance, in the last few years, several groups investigated the possible exploitation of fecal transplants. In a very interesting proof of concept study, Korpela and co-authors [70] showed the potential beneficial effects of maternal fecal transplant (MFT) on the gut microbial development of infants born by cesarean section (CS). Interestingly, they showed that the intestinal microbiota of CS children seems to be associated with a potential increased risk of developing inflammatory diseases [71]. The application of MFT to CS-born infants was shown to restore a microbiome development very similar to what is observed in vaginally born infants. Very similar results were obtained in a different context by another pilot study. Witjes and colleagues showed that fecal microbiota transplantation from vegan donors had beneficial effects in individuals with hepatic steatosis, with a trend toward improved inflammatory milieu and significant changes in expression of hepatic genes involved in inflammation and lipid metabolism [72]. Additionally, gut microbiome-derived vesicles showed on one hand to have a functional role in mediating disease [73], while on the other represent possible tools for future vaccine technologies [74], supporting the important clinical implications of this newly born field of study.

## 10. Conclusions

Despite the extensive literature on the use of EVs-related 16S detection by sequencing to assess bacterial composition and variations in several disease settings, there are still numerous questions that remain unanswered. The main issue that needs to be addressed is the source of the vesicles and, in turn, of the detected 16S. Indeed, despite the several plausible hypotheses that were made by many authors and presented in this review, no one has yet been able to define the exact origin of bacterial EVs in human fluids. Gut-leakage and release from microorganisms residing in different tissue-niches represent the most plausible sources of circulating bacterial vesicles in the physiological setting, although the presence of acute infections due to specific species can deeply affect the physiological composition of the plasma microbiome. Relative to this matter, another interesting point of discussion is whether the species detected and classified through circulating 16S sequencing represent the “whole picture” or if they are only a part of those effectively composing the human microbiota. 

Another interesting point could be represented by intracellular bacteria, which can use and modify the internal trafficking system of eukaryotic cells [75], similar to what occurs with eukaryotic pathogens [76]. In this setting, 16S molecules could be effectively vehiculated both by bacteria-released EVs and by host-cell-released vesicles with mixed prokaryotic and eukaryotic content. Anyhow, the current knowledge about the exact composition and markers of circulating OMVs is not yet complete enough to link specific vesicles with their sources. Thus, a more thorough classification of the superficial markers and cargoes characterizing EVs derived from specific bacteria represents an unmet need that could greatly improve our knowledge and possible clinical exploitability of these small “biologic shuttles”.

**Figure 2:**
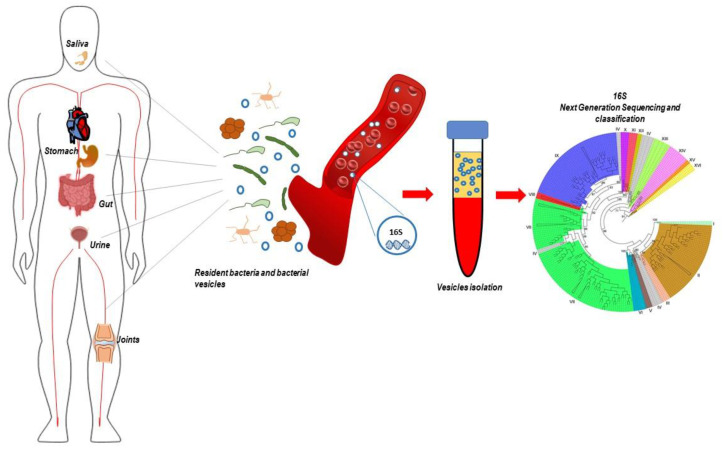
Circulating microbiome analysis. The figure depicts the proposed mechanism of classification of circulating microbiome, based on the isolation of RNA from bacteria-derived extracellular vesicles, amplification of 16S RNA, and Next-Generation Sequencing.

As presented in this review, MV-vehiculated 16S seems to be a mirror of systemic microbiome composition, its variation could be used to track the onset and progression of several pathologies, thus opening to the use of prokaryotic EVs as circulating biomarkers of diseases (Figure 2).

## Figures and Tables

**Table 1:** Extracellular vesicles-based studies investigating circulating 16S.

Manuscript Ref.	Context of the Study	Extracellular Vesicles (EV) Source	Vesicles Isolation Method	16S Regions	Sequencing Tool
[57]	Acute colitis mouse model	Small intestinal fluids, stools, and culture media	Ultracentrifugation, 200.000× *g* for 2 h at 4 °C	Unspecified	Roche 454 GS FLX Titanium
[58]	Pregnant vs. non-pregnant	Human urine	Differential centrifugation method	V1—V3	Roche 454 GS FLX
[59]	Gastric cancer vs. gastric ulcers vs. duodenal ulcers.	Human gastric juices	Differential centrifugation method	V1—V3	Roche 454 GS FLX
[60]	Alzheimer disease mouse model	Mouse blood	Differential centrifugation method	V3—V4	Illumina MySeq
[61]	Chronic rhinitis vs. allergic rhinitis vs. atopic asthma.	Human urine	Differential centrifugation method	V3—V4	Illumina MySeq
[62]	Non-tuberculous mycobacterial lung disease (NTM) and NTM + breast cancer	Human sputum	Commercial Exosome Isolation Kit	V4	Illumina MySeq
[63]	Biliary tract cancer	Human blood samples	Differential centrifugation method	V3—V4	Illumina MySeq
[64]	Bipolar disorder and major depressive disorder.	Human serum	Differential centrifugation method	V3—V4	Illumina MySeq
[65]	Ovarian cancer and benign ovarian tumors.	Human serum	Differential centrifugation method	V3—V4	Illumina MySeq
